# In-vivo dosimetry using electronic portal imaging in online-adaptive radiotherapy for rectal cancer^☆^

**DOI:** 10.1016/j.phro.2026.100990

**Published:** 2026-05-03

**Authors:** Gary Razinskas, Robert Schindhelm, Anne Richter, Johannes Kraft, Marcus Zimmermann, Jörg Tamihardja, Andrea Wittig-Sauerwein, Sonja Wegener

**Affiliations:** University Hospital Würzburg, Department of Radiotherapy and Radiation Oncology, Josef-Schneider-Str. 11, 97080 Würzburg, Germany

**Keywords:** In-vivo dosimetry, Electronic portal imaging device, Online-adaptive radiotherapy

## Abstract

**Background and purpose:**

Electronic portal imaging device (EPID)-based in-vivo dosimetry (IVD) is widely used to verify radiotherapy dose delivery and detect deviations from anatomical changes, setup uncertainties, or machine-related errors. However, cone-beam computed tomography (CBCT)-guided online-adaptive radiotherapy (oART), which re-optimizes treatment plans on-couch based on daily imaging, lacks a measurement-based quality assurance framework. This study aimed to evaluate the feasibility and accuracy of EPID-based transit IVD for CBCT-based oART.

**Materials and methods:**

EPID-based IVD data from ten patients receiving short-course oART for rectal cancer on an Ethos linear accelerator were retrospectively analyzed over 50 treatment sessions using a commercial EPID-based IVD framework. Gamma pass rates (GPR) were calculated using global gamma criteria of 3 %/3 mm, 3 %/2 mm, and 2 %/2 mm. Angular dependence and field shape effects were investigated using in-air and in-phantom measurements.

**Results:**

Across all patients and fractions, the mean GPR at 3 %/3 mm with a 20 % low-dose threshold was (95.7 ± 1.8) %, with patient-specific means ranging from 91.4 % to 98.0 %. Two sessions showed notable reductions associated with large intra-fractional gas variations. Angular analysis revealed notable GPR variations, particularly for laterally incident fields.

**Conclusions:**

EPID-based IVD was a promising quality assurance approach for verifying oART delivery, achieving higher GPRs under tighter evaluation criteria than typically reported for standard image-guided radiotherapy. These findings indicate that workflow-specific tolerance criteria should be defined to reflect the reduced anatomical variation in adaptive treatments, requiring more accurate beam modeling. Automating EPID-based IVD integration into clinical protocols will be essential for safe and efficient implementation.

## Highlights

• Novel use of portal-imaging in-vivo dosimetry for online adaptive radiotherapy.
• Gamma pass rates of (95.7 ± 1.8) % exceed those in non-adaptive radiotherapy.
• Daily adaptation reduced anatomical variation and improved plan agreement.
• Angular dependence quantified using dedicated in-air and in-phantom tests.
• Findings support defining tolerance criteria specific to adaptive workflows.

## Graphical abstract

**Figure:**
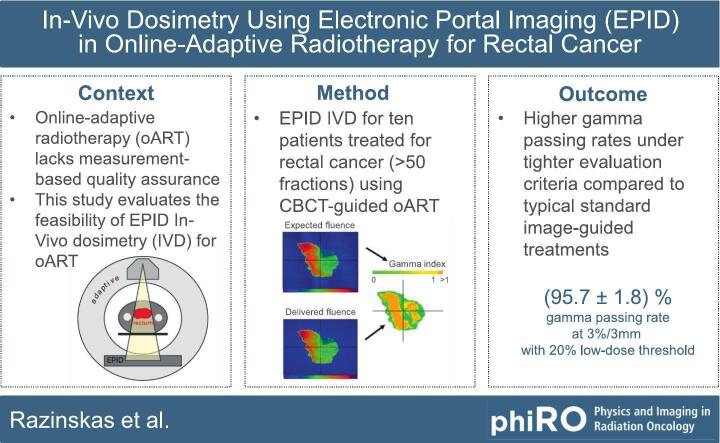

## 1 Introduction

In precision radiotherapy, the accurate delivery of the prescribed radiation dose is crucial to achieve optimal clinical outcomes while minimizing unintended exposure of surrounding healthy tissues. In-vivo dosimetry (IVD) constitutes a critical component of the quality assurance (QA) process, enabling direct verification of the dose actually delivered to the patient during treatment. IVD is defined as the measurement-based confirmation of the applied dose in or on the patient [1], enabling the detection and quantification of dose deviations that may have resulted from errors in dose calculation, patient positioning, or treatment machine settings [2], [3], [4].

Among the various IVD techniques [5], electronic portal imaging devices (EPIDs) have gained substantial recognition [6] as a powerful tool for patient-specific quality assurance (PSQA), particularly in advanced radiotherapy modalities such as intensity-modulated radiation therapy (IMRT) [7], [8], volumetric modulated arc therapy (VMAT) [2], [9], [10], and stereotactic treatments [11]. These techniques typically involve highly modulated treatment plans and high fractional doses, necessitating a comprehensive commissioning protocol for EPID-based IVD systems to ensure accuracy and reliability [12]. To achieve high dosimetric accuracy raw EPID signals are usually pre-processed to correct for dead pixels, pixel sensitivity variations, pixel-value-to-dose conversion (if applicable), and energy response [13], [14], [15]. The current amorphous silicon (a-Si) EPID technology offers high-resolution imaging, superior sensitivity, and rapid digital data acquisition, while also exhibiting favourable dosimetric characteristics. Unlike conventional point detectors, which provide only localized surface dose information, EPIDs offer the advantage of capturing direct information of the spatial dose distribution within the patient. Moreover, their potential for automation [16], [17] and applicability in both two-dimensional (2D) and three-dimensional (3D) dosimetric verification have positioned them as a preferred modality for transit IVD.

EPID-based IVD can be performed using different dose reconstruction methodologies, primarily categorized into forward and backward projection methods [18]. In forward projection the measured exit radiation is converted into a dose or fluence map at the EPID plane for comparison with the planned reference distribution. In contrast, backward projection reconstructs an estimated dose distribution within the patient, which can be directly compared to the planned dose. Both approaches offer distinct advantages in evaluating treatment accuracy [19]. In this study, we applied the forward-projection approach using a commercial platform, as it provides a practical workflow for evaluating transit images in online adaptive treatments.

The integration of EPID-based IVD into online-adaptive radiotherapy (oART) workflows is particularly compelling, as conventional measurement-based QA strategies are impractical in this setting. Although previous studies described the use of EPID-based IVD for adaptive workflows in MR-linac systems [20], [21], [22], to date, no systematic implementation has been reported for cone-beam CT (CBCT)-based oART. Given the inherent complexity of plan adaptation workflow, EPID-based IVD could serve as a vital tool for detecting and mitigating errors related to dose recalculation, patient positioning, and anatomical changes during treatment [23]. Although current data-access constraints prevent real-time or on-couch EPID-IVD, it still provides post-delivery verification that can reveal systematic deviations and guide corrections in subsequent fractions.

The aim of this study was to evaluate the feasibility, dosimetric accuracy, and angular dependence of EPID–based transit IVD for CBCT–guided oART delivered on an O-ring gantry linear accelerator. We assessed performance across clinically relevant gamma criteria using patient treatments and complementary phantom measurements to identify workflow-specific strengths and limitations. These findings were intended to support oART-specific tolerance criteria and to guide future integration of automated EPID-based IVD analysis into adaptive radiotherapy workflows.

## 2 Material and methods

### 2.1 Treatment plans and adaptive session

This study retrospectively analyzed transmission images acquired with the aS1200 EPID panel for ten patients undergoing CBCT-guided oART on an Ethos linear accelerator (Varian Medical Systems, Palo Alto, CA, USA). Eligible patients received neoadjuvant short-course radiotherapy for locally advanced rectal cancer or short-course radiotherapy as monotherapy for palliation, and were treated between May 2022 and April 2023. All patients received treatment plans prescribing a median dose of 5 Gy per fraction to the planning target volume (PTV). Eight patients were treated with a total dose of 25 Gy in five fractions, while two patients received 30 Gy in six fractions; for these latter cases, the first five fractions were included in the analysis. This single-center study was approved by the local institutional Ethics review board (2026–78-dvhd).

Treatment planning was performed in Ethos Treatment Management (version 2.1), using 9, 11, or 12 field IMRT, selected at the planners’ discretion. For each treatment session, the adapted plan was automatically proposed by the system and selected based on daily anatomical assessment.

Following each session, synthetic CT datasets and adapted treatment plans were manually exported via the Ethos software’s session review environment for subsequent dosimetric verification. EPID images, corrected by the standard aS1200 postprocessing chain (dark-field, flood-field, and dead-pixel corrections), were automatically recorded and stored by the treatment system. However, they could not be accessed by clinical staff and had to be retrieved by vendor technicians, as no automated EPID export is currently available. Thus, EPID-based IVD could only be performed post-treatment and not in real-time. Unlike conventional machine-focused QA, this study evaluated patient-specific dosimetric accuracy within a clinical oART workflow.

### 2.2 In-vivo dosimetry

All treatment sessions were imported into SunCHECK (Sun Nuclear Corporation, Melbourne, FL, USA, version 4.3.0) for dosimetric evaluation. Dose calculations utilized a vendor-customized Ethos beam model, calibrated using previously acquired square field measurements in accordance with a standardized beam characterization protocol. This calibration included vendor-supplied plans consisting of multiple square field beams delivered from a fixed 0° gantry angle: one plan was delivered in air for Fraction 0 (pre-treatment QA), and another for Fraction N (in-vivo), which was delivered twice through slab phantoms made of solid water with two different thicknesses placed in the beam path.

To assess dosimetric accuracy, absolute global Gamma pass rates (GPR) were computed by comparing EPID-measured transit fluence distributions with the corresponding calculated reference fluence distributions. These reference fluence maps were generated automatically by the SunCHECK dose-reconstruction engine from each session’s adapted plan and synthetic CT, yielding the expected EPID-level transit fluence. Gamma analysis was performed for each treatment (Fraction N) using three gamma criteria: 3 %/3 mm, 3 %/2 mm, and 2 %/2 mm, with either 10 % or 20 % low-dose thresholds. Stricter gamma criteria were used than in typical image-guided radiotherapy (IGRT) because online adaptation is expected to reduce inter-fractional anatomical uncertainty.

To analyze intra-fractional anatomical changes, all image and plan data were imported into Eclipse (v18.0, Varian Medical Systems, Palo Alto, CA, USA). Gas volumes were quantified volumetrically using manually defined structures, and dose was recalculated using the Acuros XB algorithm. To evaluate the impact of gas-related anatomical changes on target coverage dose–volume histogram metrics were extracted for the PTV and a reduced target volume PTV-0.5. PTV-0.5, defined as a uniform 0.5 cm isotropic shrink of the PTV, was used as a surrogate for the CTV to assess target coverage with reduced sensitivity to small voxel-level deviations at the PTV boundary.

### 2.3 Angular dependence analysis

Variations in GPR across different beam angles within a single treatment plan may result from two primary sources: (1) variations in field shape across angles, or (2) intrinsic angular dependence of the EPID response, gantry or portal sagging due to gravity and potential beam modeling inaccuracies. To distinguish between these effects, a dedicated experimental setup was implemented.

A 12-field IMRT plan, representative of the rectal cancer treatments in this study, was selected. To assess angular dependence, twelve additional test plans were created—one for each gantry angle of the original clinical plan. In each test plan, the original IMRT field shapes, fluence patterns, and monitor units were preserved, but all fields were delivered at the same fixed gantry angle. Thus, for every gantry angle in the original plan, a test plan was generated in which all twelve IMRT fields were delivered from that single angle, isolating the effect of gantry position while keeping field shape and modulation constant. Measurements were acquired at the discrete gantry angles of the original multi-field IMRT plan.

The test plans were delivered under two measurement conditions: in-air measurements (to isolate intrinsic angular dependence of the EPID) and in-phantom measurements using a 27 cm-diameter water-filled cylindrical phantom, consisting of a standard retail cylindrical barrel filled with water and positioned centrally at the Ethos treatment isocenter to incorporate the effects of tissue attenuation and scatter.

Dosimetric analysis was conducted using SunCHECK software by comparing EPID-measured fluences maps with their corresponding calculated fluence distributions. For all treatment fields, monitor units (MU) per field were extracted from the treatment plan data, and fluence complexity was quantified based on predicted fluence maps. Field complexity was quantified using the V_400_/P_400_ metric described by Hanušová et al. [24], where P_400_ denotes the number of pixels with dose gradients exceeding a threshold of 400 and V_400_ the sum of their gradient amplitudes. This threshold excludes small out-of-field gradients, as recommended in the original work. Testing for a correlation, Spearman’s rank correlation coefficients were calculated to assess the relationship between GPR and MU or complexity (V_400_/P_400_), respectively.

## 3 Results

### 3.1 In-vivo dosimetry

**Fig. 1:**
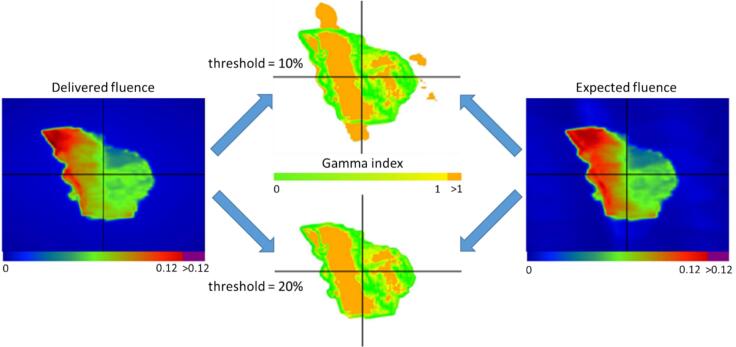
EPID-based IVD Gamma analysis comparison for different low-dose threshold. The left panel displays the delivered fluence distribution for an exemplary treatment field of a patient with rectal cancer undergoing CBCT-guided oART, while the right panel shows the corresponding expected fluence distribution, reconstructed using the forward-propagation method. The middle column shows the 2 %/2 mm gamma maps. The top row applies a 10 % low-dose threshold, revealing localized discrepancies in low-dose regions, while the bottom row uses a 20 % low-dose threshold.

Table 1 summarizes the GPR for EPID-based IVD across ten rectal cancer patients under different evaluation criteria, demonstrating consistently high agreement across patients and fractions. Visual analysis of Gamma pass/fail points at a 10 % low-dose threshold showed localized discrepancies associated with low-dose regions (Fig. 1). Using a 20 % low-dose threshold, these low-dose regions were excluded from the evaluation, and the corresponding artifacts were no longer visible in the Gamma analysis. Nevertheless, overall GPR values remained comparable irrespective of the threshold applied (Table 1). A strong linear correlation (R^2^ = 0.99) was observed between GPR values obtained with 10 % and 20 % low-dose threshold, indicating that both settings provide highly consistent results.

**Table 1:** GPR for IVD under different evaluation criteria. Mean absolute GPR across all treatment fields and sessions per patient are shown for three gamma criteria (3 %/3 mm, 3 %/2 mm, and 2 %/2 mm) with 10 % and 20 % low-dose threshold. The average GPR across all patients A-K is provided along with one standard deviation (SD) of these patient-specific means.

	GPR [%]					
	10 % low-dose threshold			20 % low-dose threshold		
Patient	3 %/3 mm	3 %/2 mm	2 %/2 mm	3 %/3 mm	3 %/2 mm	2 %/2 mm
A	97.5	88.4	81.1	97.1	87.7	80.2
B	95.0	83.1	75.5	95.0	83.0	74.9
C	95.4	86.1	79.7	95.4	86.7	80.6
D	96.0	82.7	73.6	95.5	82.6	73.5
E	97.7	90.5	84.9	97.7	90.3	84.3
F	95.4	76.4	64.5	95.6	77.8	66.3
G	95.6	85.2	76.7	95.3	85.0	75.3
H	91.8	70.2	59.6	91.4	69.7	59.5
I	97.4	87.6	79.6	98.0	87.8	79.4
K	95.7	80.9	72.0	96.1	80.8	71.6

Average	95.8	83.1	74.7	95.7	83.1	74.6
1 SD	1.7	6.1	7.7	1.8	6.0	7.4

**Fig. 2:**
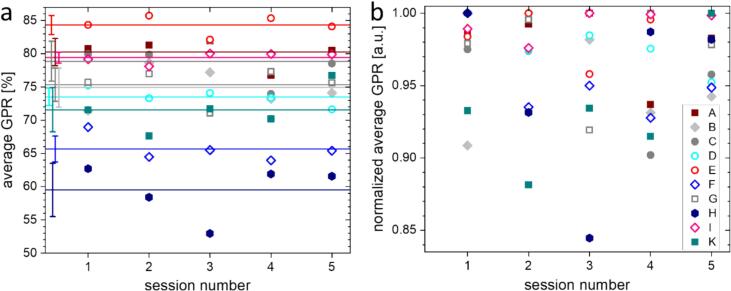
Session-specific in-vivo GPR for CBCT-guided oART. (a) Mean IVD GPR (2 %/2 mm, 20 % low-dose threshold) per session, averaged over all treatment fields for each patient. The horizontal line represents the mean GPR across all sessions for a given patient, with error bars indicating one standard deviation (SD). (b) Mean IVD GPR per session normalized to the maximum average GPR per patient. This panel highlights inter-session variations in GPR within individual patients.

Fig. 2a shows the session-specific mean GPR (2 %/2 mm, 20 % low-dose threshold) across all fields within each treatment plan for all patients. Inter-patient variability was evident, with Patient E exhibiting the highest mean GPR (84.3 ± 1.4) % and Patient H the lowest (59.5 ± 4.0) %, while Patient H also showed the largest intra-patient variation, reflected by the highest standard deviation (4.0 %). Two outlier sessions were identified: Session 2 of Patient K and Session 3 of Patient H. Both sessions exhibited notably lower GPR values compared to other fractions (Fig. 2b).

Further analysis of two outlier sessions (Session 2 of Patient K, Session 3 of Patient H) revealed that lower GPR values were associated with pronounced intra-fractional anatomical changes. In Patient K for example, approximately 23 cm^3^ of gas was observed within or adjacent to the PTV on the initial CBCT, increasing to 27 cm^3^ during the treatment session. In contrast, Session 5 for the same patient, which exhibited the highest GPR, contained negligible gas within the PTV except for one gas pocket (<1 cm^3^) in the post-treatment CBCT. Similarly, in Patient H, gas volume near the PTV rose from 33 cm^3^ to 46 cm^3^ and then decreased slightly to 40 cm^3^ post-treatment. These anatomical changes, while influencing the EPID-measured transit fluence, only led to modest dosimetric deviations in PTV coverage, with changes in PTV–0.5 D_98  %_ limited to –0.3 % and +0.7 % for Patients K and H, respectively.

**Fig. 3:**
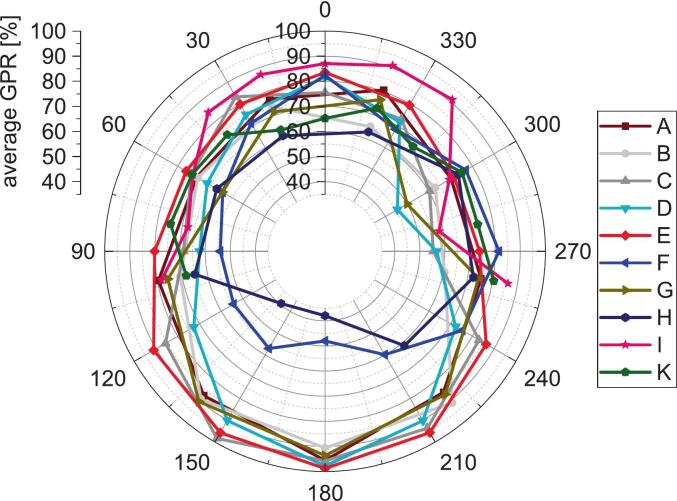
Angular dependence of in-vivo GPR for rectal cancer treatments: Mean IVD GPR (2 %/2 mm, 20 % low-dose threshold) for each treatment field of the IMRT treatment plan, averaged across all sessions for every patient. The data is displayed in a polar plot, where the angle represents the incident gantry angle of the treatment field. Markers are connected with lines for visual aid.

Within individual treatment plans, field-specific GPR values showed a dependence on gantry angle (Fig. 3). For fields delivered at a gantry angle of 0°, the mean GPR was approximately 79 %, whereas posterior fields delivered at 180° exhibited higher mean GPR values of approximately 89 %. No systematic temporal trend was observed across treatment fractions, with GPR values remaining stable over the course of treatment. The angular GPR distribution was approximately symmetric with respect to the vertical axis, as illustrated by the polar representation in Fig. 3.

### 3.2 Angular dependencies in the absence of a patient

**Fig. 4:**
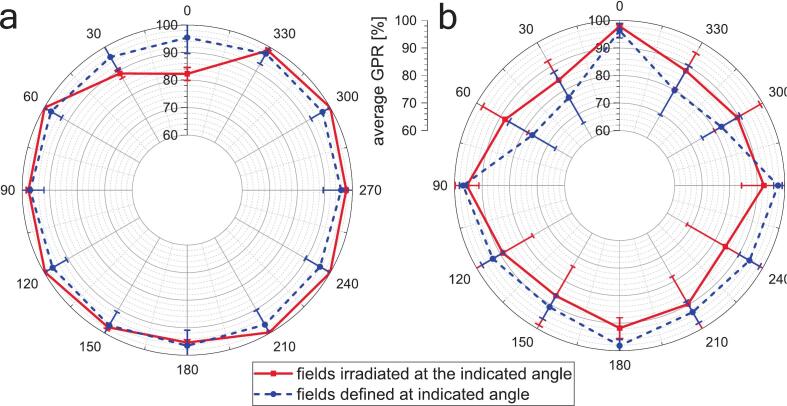
(a) In-air (Fraction 0) and (b) in-phantom (Fraction N) GPR (2 %/2 mm, 20 % low-dose threshold) for the representative 12-field test plans evaluated at discrete beam angles. Markers indicate the discrete measurement points, which are connected by lines for visual aid. For both panels, the red solid line represents the mean GPR for all 12 field shapes irradiated at each specified beam angle, while the blue dashed line shows the mean GPR for a fixed field shape defined at the indicated angle and delivered from all 12 beam angles. (For interpretation of the references to colour in this figure legend, the reader is referred to the web version of this article.)

Fig. 4 summarizes angular-dependent GPR behavior under idealized conditions without patient anatomy, based on in-air (Fraction 0) and in-phantom (Fraction N) measurements. For in-air measurements, GPR values dropped for fields delivered at 0° and 30° gantry angles (Fig. 4a, red solid line). A stripe-like artifact pattern was observed in gamma maps, with failing points predominantly localized at multileaf collimator (MLC) leaf edges, suggesting that MLC positioning accuracy may contribute to the observed deviations.

Conversely, in-phantom measurements using the water-filled cylindrical phantom showed higher GPR for fixed fields at cardinal angles (0°, 90°, 180°, and 270°) compared to intermediate angles (Fig. 4b, blue dashed line). Gamma failures in the phantom-based analysis were typically located at MLC leaf tips, sometimes forming stripe patterns perpendicular to those observed in the in-air measurements.

A statistical analysis of fields exhibiting GPR (2 %/2 mm, 20 % low-dose threshold) values below 80 % revealed that these fields often corresponded to fields characterized by a high number of monitor units per control point and low fluence complexity (V_400_/P_400_). However, Spearman’s rank correlation coefficients of 0.45 and 0.41 (not statistically significant) indicate only a moderate association. This suggests that additional factors may contribute to the angular-dependent variations observed in GPR.

## 4 Discussion

This study evaluated EPID-based IVD in CBCT-based oART for rectal cancer. Across 50 sessions, high GPR were achieved under strict criteria, with remaining deviations mainly attributable to intra-fractional anatomical changes. These results support the feasibility of integrating EPID-based transit dosimetry into adaptive workflows.

The results demonstrated that stringent gamma analysis criteria (e.g., 3 %/3 mm) yielded high global GPR, with an average of 95.7 % across all patients and treatment fields (Table 1). These findings were consistent with acceptance thresholds proposed in AAPM Task Group Report 307 [3], [25]. While the most conservative tolerances limits reported across various anatomical sites use 3 %/3 mm global gamma with a 90 % acceptance threshold [26], [27], more lenient criteria (e.g., 5 %/5 mm, 20 % low-dose threshold) are often applied in clinical practice. Comparable EPID-based IVD studies in non-adaptive rectal and pelvic radiotherapy consistently reported lower agreement despite using looser criteria. Esposito et al. [28] found notable out-of-tolerance rates in a large multicenter cohort even with relaxed criteria; Peca et al. [29] identified frequent delivery deviations in standard rectal radiotherapy due to anatomical and setup variability; and Bossuyt et al. [30] reported a 93 % acceptance threshold for rectal cancer plans using 5 %/5 mm criteria.

In contrast, all oART sessions in this study met or exceeded 3 %/3 mm global gamma acceptance criteria or mean gamma pass rates reported in the literature. The high GPR values reflect the inherent advantages of oART: daily imaging and on-couch plan adaptation effectively eliminate inter-fractional anatomical variations, which is known to compromise the sensitivity of 3D transit dosimetry [25]. Consequently, IVD in the adaptive radiotherapy setting should be evaluated using more stringent, site-specific criteria rather than adopting thresholds from conventional IGRT.

Reduced GPR values observed in the identified outlier sessions were associated with pronounced intra-fractional anatomical changes, particularly variations in rectal gas volume. In contrast, improved agreement in sessions with negligible intra-fractional gas likely reflects a closer anatomical match between CBCT and planning CT, thereby reducing sensitivity to known limitations of the Ethos deformation-based sCT generation, as reported in recent studies [31], [32]. These findings indicate that reduced GPRs primarily reflect intra-fractional anatomical alterations rather than true dose delivery errors. However, such discrepancies warrant further investigation, particularly if target coverage may be affected.

Although overall GPR values were higher for oART than typically reported for standard IGRT IVD, certain fields—particularly those delivered at lateral angles or 0° gantry position—exhibited lower pass rates (Fig. 3). This angular dependency was not present in in-air measurements (Fraction 0, Fig. 4a), suggesting a combination of potential beam modeling inaccuracies and patient-specific anatomy and scatter effects. Interestingly, discrepancies emerged between in-air (Fig. 4a) and in-phantom (Fig. 4b) gamma analyses, which may reflect differences in dose calculation algorithms or limitations in the generic beam model used for reconstruction. These discrepancies were not apparent when looser gamma tolerances were applied, reinforcing the need for workflow-specific tolerance thresholds in adaptive QA protocols.

The findings of this study demonstrated the feasibility of EPID-based IVD for CBCT-guided oART and highlight its potential to detect intra-fractional anatomical variations that may go unnoticed with standard QA methods, e.g. secondary dose calculation. However, routine clinical integration is limited by the lack of automated EPID data access. EPID images currently require vendor-only retrieval, preventing workflow integration and making time and resource estimates impractical. Improved system-level data accessibility will be essential before EPID-based IVD can be reliably adopted in daily adaptive practice.

As a proof-of-concept, this study is subject to several limitations. The small sample size of ten rectal cancer patients restricts statistical generalizability and may not capture the full range of anatomical variability. Furthermore, all treatment plans applied homogeneous PTV dose prescriptions without simultaneous integrated boosts (SIB), meaning the evaluation was limited to cases with relatively low fluence complexity. Moreover, no systematic validation of the EPID-based IVD system in non-adaptive IGRT was performed, preventing a direct comparison of accuracy and workflow-specific gamma criteria between adaptive and conventional settings. The applicability of this approach to more anatomically complex sites—such as cervical cancer, where target deformation is more pronounced—has not yet been systematically explored. A full angular-dependence evaluation using simple, non-modulated fields should be included in future studies to better isolate beam-model and EPID-response effects.

Future investigations should aim to validate this workflow across a broader range of tumor sites, more complex dose concepts (e.g., multi-level SIB), and highly modulated plans. Moreover, insights gained from this study may guide the refinement of EPID calibration routines, including adaptive corrections based on observed deviations. In parallel, the development of adaptive-specific tolerance criteria and fully automated EPID-based IVD analysis pipelines will be essential for enabling efficient and reliable integration of this QA strategy into clinical practice.

In summary, this study evaluated the feasibility of 2D transit EPID-based IVD for CBCT-guided online-adaptive radiotherapy across 50 treatment sessions in ten patients. EPID-based IVD proved to be a viable and effective tool for integrating measurement-based QA into adaptive workflows. The exclusion of inter-fractional anatomical variations through daily CBCT-guided plan adaptation resulted in consistently high GPR and supported the application of stricter evaluation criteria compared to conventional IGRT. These findings underscore the need for dedicated tolerance thresholds tailored to the unique features of adaptive radiotherapy. Future work should aim to automate EPID-based IVD analysis and establish standardized clinical thresholds to facilitate its routine implementation in adaptive radiotherapy workflows.

## CRediT authorship contribution statement

**Gary Razinskas:** Writing – review & editing, Writing – original draft, Methodology, Investigation, Formal analysis, Conceptualization. **Robert Schindhelm:** Writing – review & editing, Investigation, Formal analysis. **Anne Richter:** Writing – review & editing, Investigation, Formal analysis. **Johannes Kraft:** Writing – review & editing, Investigation, Formal analysis. **Marcus Zimmermann:** Writing – review & editing, Investigation, Formal analysis. **Jörg Tamihardja:** Writing – review & editing, Investigation, Formal analysis. **Andrea Wittig-Sauerwein:** Writing – review & editing, Investigation, Formal analysis. **Sonja Wegener:** Writing – review & editing, Writing – original draft, Methodology, Investigation, Formal analysis, Conceptualization.

## Declaration of competing interest

The authors declare that they have no known competing financial interests or personal relationships that could have appeared to influence the work reported in this paper.
